# Kawasaki disease (KD) and multisystem inflammatory syndrome in children (MIS-C) in a Middle Eastern patient cohort

**DOI:** 10.1186/s12969-023-00834-7

**Published:** 2023-06-29

**Authors:** Lemis Yavuz, Sarmad AlHamdani, Samah Alasrawi, Deena Wafadari, Ali Al-Fraihat, Marwa A. Bebars, Jaidev Nath, Diego Arango, Marco Pallavidino, Ruchi Jain, Sinan Yavuz, Mohamed AlAwadhi, Abdulla Alkhayat, Ahmad Abou Tayoun, Walid Mohammad Abuhammour

**Affiliations:** 1 Al Jalila General Pediatric Department, Al Jalila Children’s Hospital, Dubai, United Arab Emirates; 2 Al Jalila Infectious Disease Department, Al Jalila Children’s Hospital, Dubai, United Arab Emirates; 3 Al Jalila Genomics Center, Al Jalila Children’s Hospital, Dubai, United Arab Emirates; 4 Al Jalila Children’s Heart Center of excellence, Al Jalila Children’s Hospital, Dubai, United Arab Emirates; 5 Al Jalila Pediatric Intensive Care Unit, Al Jalila Children’s Hospital, Dubai, United Arab Emirates; 6 Al Jalila Residency Program, Al Jalila Children’s Hospital, Dubai, United Arab Emirates; 7 Pediatric Department, Dubai Hospital, Dubai, United Arab Emirates; 8 Al Qasimi General Pediatric Department, Al Qasimi Women and Children’s Hospital, Sharjah, United Arab Emirates; 9 Center for Genomic Discovery, Mohammed Bin Rashid University of Medicine and Health Sciences, Dubai, United Arab Emirates; 10 Al Jalila Children’s Specialty Hospital, Al Jaddaf Street, PO Box 7662, Dubai, 7662 United Arab Emirates

**Keywords:** KD, MIS-C, Pediatrics, Fever in children, Inflammatory disease, COVID-19

## Abstract

**Objective:**

This is a comprehensive characteristic study of Kawasaki disease (KD) and Multi system inflammatory syndrome in children (MIS-C) in the Middle East that creates a formula to differentiate between the two.

**Methods:**

We conducted a descriptive comparative study of KD and MIS-C in the United Arab Emirates. Retrospective MIS-C and KD cohorts were recruited between January 2017 until August 2021.We compared clinical and laboratory characteristics between both groups. Our data were compared with 87 patients with KD or MIS-C from the literature.

**Results:**

We report on123 patients. Sixty-seven (54%) met the criteria for KD (36 male, 43 Arab), and fifty-six (46%) met the criteria for MIS-C (28 male, 35 Arab). The median age was 2.2 years range (0.15–10.7) in the KD group and 7.3 years (0.7–15.2) in the MIS-C group (P < 0.001). The clinical features on admission showed an increase in gastrointestinal manifestations in MIS-C compared with KD (84% vs. 31%, P < 0.001). Laboratory tests on admission revealed a significant increase in the following tests in KD compared with MIS-C; white blood cells (mean 16.30 10^(3)^ µcL vs. 11.56 10^(3)^ µcL, P < 0.001), absolute neutrophils (mean 10.72 10^(3)^ µcL vs. 8.21 10^(3)^ µcL, P 0.008), absolute lymphocytes (mean 3.92 10^(3)^ µcL vs. 2.59 10^(3)^ µcL, P 0.003), erythrocyte sedimentation rate (mean 73 mm/hr vs. 51 mm/hr, P < 0.001) and platelets (median {390 10^(3)^ µcL vs. 236 10^(3)^ µcL, P < 0.001}). In contrast, procalcitonin and ferritin were increased in the MIS-C group (2.4 )ng/mL, 370 ng/mL; P **<** 0.001). Cardiac dysfunction and admission to the pediatric intensive care unit were higher in MIS-C than in KD (21% vs. 8% and 33% vs. 7.5%, respectively, P < 0.001).

**Conclusion:**

This study showed vast similarities between KD and MIS-C, suggesting that they lie along the same clinical spectrum. However, there are several differences between the two disease entities suggesting that MIS-C most likely represents a new severe variant of KD. Based on our findings in this study, we created a formula to differentiate between KD and MIS-C.

**Supplementary Information:**

The online version contains supplementary material available at 10.1186/s12969-023-00834-7.

## Introduction

Kawasaki disease (KD) is an acute febrile illness of early childhood. It is one of the most common forms of childhood vasculitis and may lead to coronary arterial complications [1]. The prognosis relies on the extent and severity of the cardiac disease. KD is a challenging disease due to unknown specific etiology, a broad spectrum of clinical manifestations and a lack of diagnostic tests. A few studies have suggested viral infection as a predisposing factor for Kawasaki disease [2].

Interestingly, during the COVID-19 pandemic, there was an increased incidence of Kawasaki-like illness related to COVID-19 infection called multisystem inflammatory syndrome in children (MIS-C) [3].

The criteria for typical Kawasaki disease include fever for five days or more in addition to the presence of four out of the five clinical presentations (extremity changes, oropharyngeal changes, nonexudative conjunctivitis, polymorphous rash, and unilateral cervical lymphadenopathy) [4]. However, in a few cases, the diagnosis can be made on the third or fourth day of fever when the patient fully met the remaining criteria, and no other etiology could explain the illness. The diagnosis of incomplete KD includes five days of fever with at least two of the clinical criteria, supported by laboratory findings, including high C-reactive protein (CRP) and a high erythrocyte sedimentation rate (ESR), in addition to three of the following findings: hypoalbuminemia, anemia, elevated liver enzymes, thrombocytosis, and leukocytosis [5]. Multisystem inflammatory syndrome in children MIS-C can be made in any patient younger than 21 years old who required hospitalization due to multiorgan involvement (at least two organs) and met the criteria of the CDC/WHO by having fever, elevated inflammatory markers, and evidence of recent COVID-19 infection confirmed either by polymerase chain reaction (PCR), COVID-19 antibodies or recent exposure to a confirmed case of COVID-19 [6, 7].

To better understand both diseases, we conducted a study in which we described the similarities and differences between them. Additionally, we created a formula to differentiate between the two.

## Methods

This is a retrospective chart review study from January 2017 until August 2021. The patients were recruited from two pediatric tertiary centers in the United Arab Emirates: Al Jalila Children’s Hospital and Dubai Hospital. The cases were identified by checking the diagnosis or discharge code. Data were utilized from the electronic medical record system and stored anonymously on password-protected computers accessed by the principal investigator.

We compared the clinical features of the two groups, KD and MIS-C.

The clinical features on admission included classical manifestations of KD in addition to gastrointestinal (GI) and neurological symptoms. Neurological symptoms were defined as headache, papilledema, blurred vision, and meningitis with or without MRI abnormalities. GI manifestation was defined as vomiting, diarrhea or abdominal pain. We calculated the percentage of cases that had positive clinical findings from the total number of patients in each group, and then we calculated the chi-square test considering a P value < 0.05 to be statistically significant.

The main characteristics of the cardiac findings were cardiac function, mitral regurgitation (MR), coronary diameter with Z score, the presence or absence of aneurysm and pericardial effusion. Cardiac dysfunction was defined when the ejection fraction was less than 55% with/without atrial or ventricular dilatation. Regarding the Z score and coronary diameter, we compared the percentage of patients who had Z scores +/- 2.5 mm and coronary artery diameters greater than 2.5 mm.

Additionally, we reviewed literature for available data of KD and MIS-C. We collected a control group of 87 patients (MIS-C = 42, KD = 45) from literature. The following variables, Age, leukocyte count and platelet count were collected for both groups. (Supplementary Tables 1 and 2). Analyzing our data revealed significant differences in the same variables between KD and MIS-C. We used those attributes from our study to build a formula that can distinguish between both. We tested this formula on the group from literature.

### Study enrollment

Patients in the study were diagnosed by their caring physicians according to the criteria of KD (typical and incomplete) specified by American Heart Association (AHA) or MIS-C specified by the Centers of Disease Control and Prevention (CDC), World Health Organization (WHO). 4,5,6,7.

### Patient population

The age group in this study was from 0 to 18 years old. Patients were divided into two groups. Group KD refers to patients who met the criteria for complete or incomplete Kawasaki disease, and the second group refers to MIS-C patients. The exclusion criteria included patients with additional etiology that could explain their illness or could interfere with the severity, such as a congenital heart disease, genetic and metabolic diseases, immunodeficiency, or other comorbidities related to chronic illnesses like chronic lung disease.

Deidentified electronic medical records were utilized to extract patient information. All data on admission were recorded, including age, sex, nationality, date of diagnosis, length of hospital stay, medical history, physical examination, laboratory test result, echocardiographic findings, and interpretation. The course of illness was also documented, including the management plan and treatment. Repeat inflammatory markers and echocardiograms, which were performed within one month of discharge, were recorded.

### Statistics

Statistical analysis was performed using SPSS version 20. We summarized categorical variables as percentages, and we used the chi-square test when the expected count was more than five. Otherwise, we used Fisher’s exact test. We summarized continuous variables as the mean (SD) and used independent group t tests when values were normally distributed; otherwise, we used the Mann‒Whitney U test with the median (IQR and range). We considered a two-sided α less than 0.05 to be statistically significant.

### Bias: Measures to minimize bias

The data abstractors were blinded to the study objectives and research questions. This was done to reduce the observer and data abstractor bias in the study.

#### IRB

This study was approved by the institutional review board of DHCR (Dubai Healthcare City Authority–Regulatory Research Department) under approval number AJCH-049.

## Results

### Demographic distribution of KD and MIS-C

In our study, we included a total of 123 patients. Sixty-seven (54%) met the criteria for KD, and fifty-six (46%) met the criteria for MIS-C. In Table 1, we compared the demographic characteristics of both groups. The KD patients were significantly younger than the MIS-C patients (P < 0.001). The age (median and IQR/year) was 2.2 years and 3.1years in the KD group and 7.3 years and 7.2 years in the MIS-C group, respectively. The percentage of males to females was 54–46% in the KD group and 50–50% in the MIS-C group (P = 0.6). For race, we divided each group into Arab and non-Arab patients. The percentage of Arab and non-Arab patients was 64% and 36% for KD and 63% and 37% for MIS-C (P = 0.8).

**Table 1:** Demographic characteristics

	KD	MIS-C	
No, (%) of total patients	67 (54)	56 (46)	
			**P-value**
Age, Median, IQR, (range)/ y	2.3, 3.1 (0.15–10.7)	7.3, 7.2 (0.7–15.2)	**< 0.001**
Male, No (%)	36 (54)	28 (50)	0.68
Female, No (%)	31 (46)	28 (50)	0.68
Ethnicity,			
Arab; {No (%)} Non-Arab; {No (%)}	{43 (64)} {24 (36)}	{35 (63)} {21 (37)}	0.85

Abbreviation: MIS-C, multisystem inflammatory syndrome in children; KD, Kawasaki Disease; No, Number of patients; IQR, interquartile range; y, years

Non-Arab; Afghanistan, American, Azerbaijan, Canadian, Dominican, Greek, Indian, Iranian, Japan, Kazakhstan, Nigeria, Pakistan, Philippine, Turkey

### Clinical features on admission

The results showed that the duration of fever on the day of diagnosis was significantly longer in the KD group (median: 6 days, IQR: 4, range 4–15) than in the MIS-C group (median: 5 days, IQR: 3, range 2–14) (P < 0.001).

**Fig. 1:**
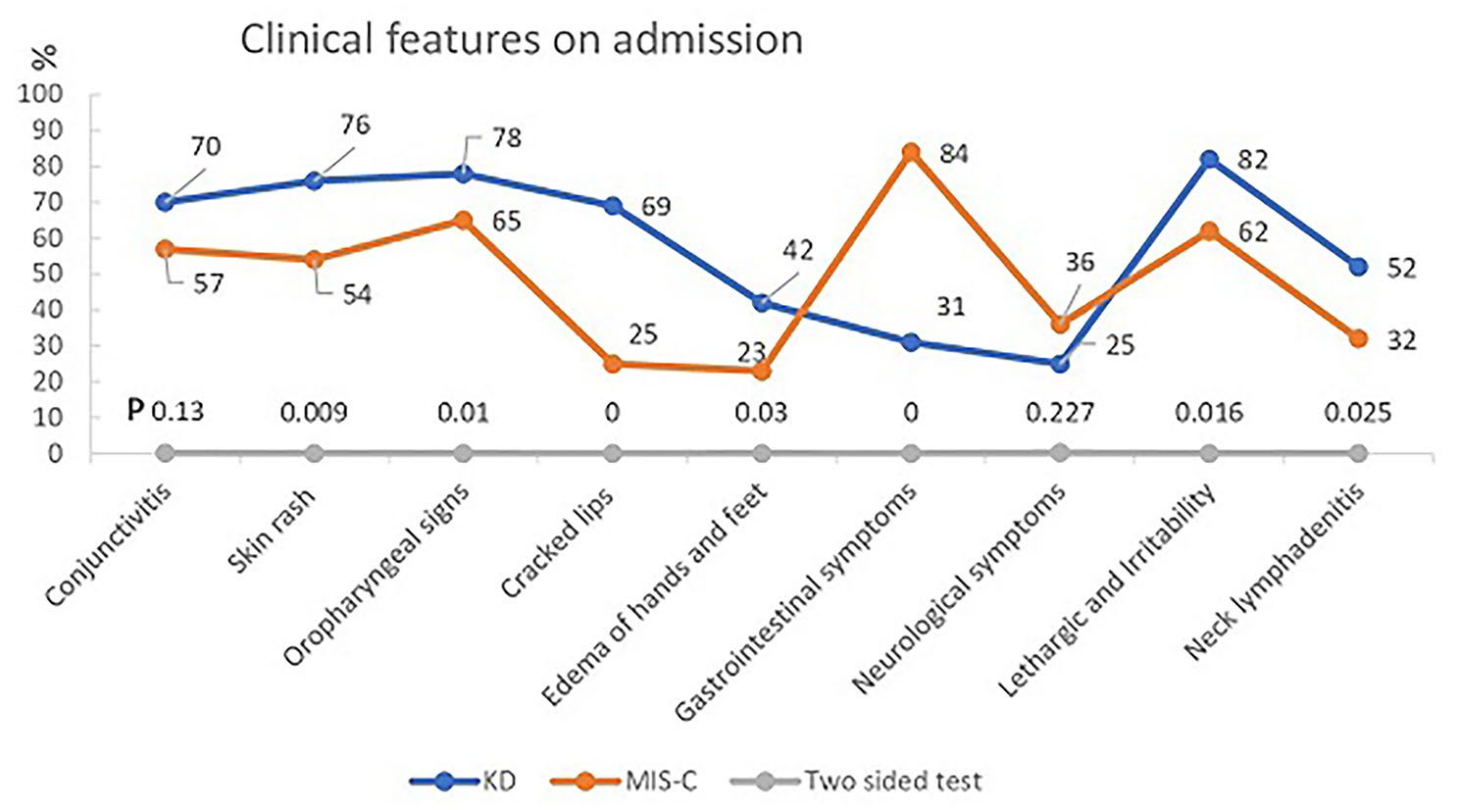
Clinical presentations on admission

There were no significant differences between the two groups in conjunctivitis, skin rash, or neurological symptoms. There was a significant difference in oropharyngeal signs, cracked lips, edema of the hands and feet, neck lymphadenitis, lethargy, and irritability (P < 0.05). These features tended to be more common in the KD group than in the MIS-C group. Interestingly, gastrointestinal symptoms were less prevalent in the KD group (31%) than in the MIS-C group (84%) (P < 0.001) (Fig. 1).

### Laboratory results on admission and 2–4 weeks after illness

Table 2 shows that the mean white blood cell count (WBC), absolute neutrophil count (ANC) and absolute lymphocyte count (ALC) were significantly higher ( P < 0.001, P = 0.008, P = 0.003 respectively) in the KD group (16.30 10^(3)^ µcL, 10.72 10^(3)^ µcL, and 3.92 10^(3)^ µcL, respectively), than in the MIS-C group (11.56 10^(3)^ µcL, 8.21 10^(3)^ µcL, and 2.59 10^(3)^ µcL, respectively). The platelet count (PLT) increased significantly (P < 0.001) in KD (median: 390,000 10^(3)^ µcL) compared with MIS-C (median: 236,000 10^(3)^ µcL). Additionally, the mean ESR level in KD patients (73 mm/hr) was significantly higher than that in MIS-C patients (51 mm/hr) (P < 0.001). Additionally, there were significant differences in the levels of procalcitonin (PCT) and ferritin, which were lower in the KD group (median: 0.15 ng/mL and 195 ng/mL, respectively) than in the MIS-C group (median: 2.4 ng/mL and 370 ng/mL, respectively) (P < 0.001). The renal function results showed increased creatinine levels in MIS-C patients (mean: 0.44 mg/dL) compared with KD patients (mean: 0.27 mg/dL) as well as blood urea nitrogen (median: 22 mg/dL in MIS-C patients and 18 mg/dL in KD patients, P < 0.05). There were no significant differences in the rest of the laboratory results between the two groups, including hemoglobulin Hb, C-reactive protein (CRP), alanine aminotransferase (ALT), aspartate aminotransferase (AST), albumin (ALB), D-dimer, and fibrinogen or in the urine analysis, including WBCs, red blood cells (RBCs) and protein.

**Table 2:** Laboratory findings on admission

	KD					MIS-C				Normal range (unit)		Two tailed T-Test
	No	Mean		SD		No	Mean		SD			
WBC	67	16.30		5.56		56	11.56		5.97	(5–15) 10^(3)^ µcL		**< 0.001**
Hb	67	10.57		1.39		56	10.73		2.06	(11–14) mg/dL		0.62
ANC	67	10.72		5.0		56	8.21		5.2	(1–8) 10^(3)^ µcL		**0.008**
ALC	67	3.92		2.5		56	2.59		2.1	(4–9) 10^(3)^ µcL		**0.003**
ESR	56	73		32		50	51		28	(< 10) mm/hr		**< 0.001**
Creatinine		0.27		0.11			0.44		0.21	(0.1–0.36) mg/dL		**< 0.001**
	**No**	**Median (range)**		**IQR**		**No**	**Median (range)**		**IQR**			
PLT	63	390 (125–1136)		205.5		56	236 (38–209)		209	(200–490) 10^(3)^ µcL		**< 0.001**
CRP	66	108 (10–391)		55		55	126 (7-492)		158	(0-2.8) mg/dL		0.38
PCT	39	0.15 (0.00–54)		0.99		50	2.4 (0.12–99.5)		9.8	(0-0.5) ng/mL		**< 0.001**
ALT	60	28 ((7-396)		28		53	34 (5-798)		45	(0–39) U/L		0.38
AST	37	34 (13–296)		24		50	34 (9-462)		31	(0–51) U/L		0.79
ALB	61	3.5 (2.1–4.4)		0.60		51	3.4 2-4.3)		2.3	(3.8–5.4) g/dL		0.44
Urea	49	18 (9–66)		9		49	22 (3-103)		13	(19–47) mg/dL		**0.029**
Ferritin	27	195 (42-4977)		241		53	370 (64-1688)		488	(6–67) ng/mL		**< 0.001**
D-dimer	10	2.4 (0.79–4.7)		2.5		55	2.06 (0.17–11.8)		2.9	(0-0.5) ug/mL		0.73
Fibrinogen	13	519 (333–900)		239		45	522 (191–864)		192	(162–401) mg/dL		0.55
	**No**	**(%)**				**No**	**(%)**					
Urine WBC	41	18				45	12			(0–5)/hpf		0.09
Urine RBC	52	14				42	24			(0–5)/hpf		0.19
Urine Protein	52	31				46	39			> +		0.38

Abbreviation: No, Number of patients who have data; WBC, White Blood Cells; Hb, Haemoglobin; ANC; Absolute Neutrophils; ALC, Absolute lymphocyte; PLT, Platelet; CRP, C-Reactive Protein; ESR, Erythrocyte Sedimentation Rate; PCT, Procalcitonin; ALT, Alanine Aminotransferase; AST, Aspartate Aminotransferase; ALB, Albumin; Urine WBC, white blood cell in urine more than 5; Urine RBC, red blood cell in urine more than 5; Urine protein, proteinuria; KD, Kawasaki disease; MIS-C, Multi system inflammatory syndrome in children, SD; standard deviation

Repeat inflammatory markers 2–4 weeks after treatment showed normalized values without significant changes between both groups regarding WBC, Hb, ANC, CRP, PCT and ESR. Interestingly, the absolute lymphocyte count remained significantly lower in the MIS-C group (median: 3.9 10^(3)^ µcL) than in the KD group, (median: 5.5 10^(3)^ µcL) (P = 0.03), while ALT was higher in the MIS-C group (median: 26.5 U/L; range: 11–412) than in the KD group (median: 17.5 U/L; range: 8–39) (P = 0.008) (Table 3).

**Table 3:** Laboratory findings in two to four weeks

	KD					MIS-C				Two tailed T-Test
	No	Mean		SD		No	Mean		SD	
WBC	32	11.18		3.7		33	12.7		6.6	0.85
Hb	41	11		1.2		34	10.7		1.98	0.43
AST	10	33		14		23	36		16	0.60
ALB	14	4		0.37		27	3.8		0.54	0.13
	**No**	**Median (range)**		**IQR**		**No**	**Median (range)**		**IQR**	
ANC	30	3.6 (1.01–14.2)		2.5		34	4.46, (1.09–23.09)		7.05	0.09
ALC	34	5.5, (1.9–12.6)		3.6		34	3.9 (0.7–11.8)		5.2	**0.03**
PLT	41	451 (262–1270		208.5		34	370 (172–1236)		258	0.05
CRP	20	4 (1-190)		7		31	16.4 (1–72))		15	0.09
ESR	19	15 (2-140)		33		23	19 (7–95)		29	0.43
ALT	14	17.5 (8–39)		8		26	26.5, (11–412)		44	**0.008**

Abbreviation: No, Number of patients who have data; WBC, White Blood Cells; Hb, Haemoglobin; ANC; Absolute Neutrophils; ALC, Absolute lymphocyte; PLT, Platelet; CRP, C-Reactive Protein; ESR, Erythrocyte Sedimentation Rate; ALT, Alanine Aminotransferase; AST, Aspartate Aminotransferase; ALB, Albumin; KD, Kawasaki disease; MIS-C, Multi system inflammatory syndrome in children

### Cardiac findings

On admission, the prevalence of cardiac dysfunction was significantly higher (P < 0.001) in the MIS-C group (21%) than in the KD group (8%). In addition, the coronary diameter was higher in the MIS-C group than in the KD group, as 71% of patients with MIS-C had a coronary diameter > 2.5 mm (compared with 51% of patients with KD) (P = 0.05).

The other cardiac findings were similar in both groups, with no significant differences. A repeat echocardiogram at 2–4 weeks showed improved cardiac function in the MIS-C group without substantial changes in all other cardiac parameters between the groups (Table 4).

**Table 4:** Cardiology findings on admission and in 2–4 weeks

	KD		MIS-C		P- Value
	No	%	No	%	
Aneurysm on admission	65	3	54	4	0.85
Aneurysm in 1 month	37	3	31	0	1
Coronary diameter on admission > 2.5 mm	51	51	35	71	0.05
Coronary diameter in 1 month > 2.5 mm	37	40	21	54	0.32
Coronary artery Z-Score on admission ± 2.5	37	40	30	37	0.76
Coronary artery Z-Score ± 2.5 in 1 month	26	35	20	24	0.08
MR on admission	67	30	56	30	0.95
MR in 1 month	37	19	31	22	0.49
Cardiac dysfunction on admission	67	8	56	21	**< 0.001**
Cardiac dysfunction in 1 month	37	0	34	3	0.47
Pericardial effusion on admission	67	9	56	20	0.08
Pericardial effusion in 1 month	37	8	31	10	1

Abbreviation: In 1 month, in 2–4 weeks; MR, mitral regurgitation; No, total number of patients with data; %, percentage of patients with positive findings

### Treatment and outcome

**Fig. 2:**
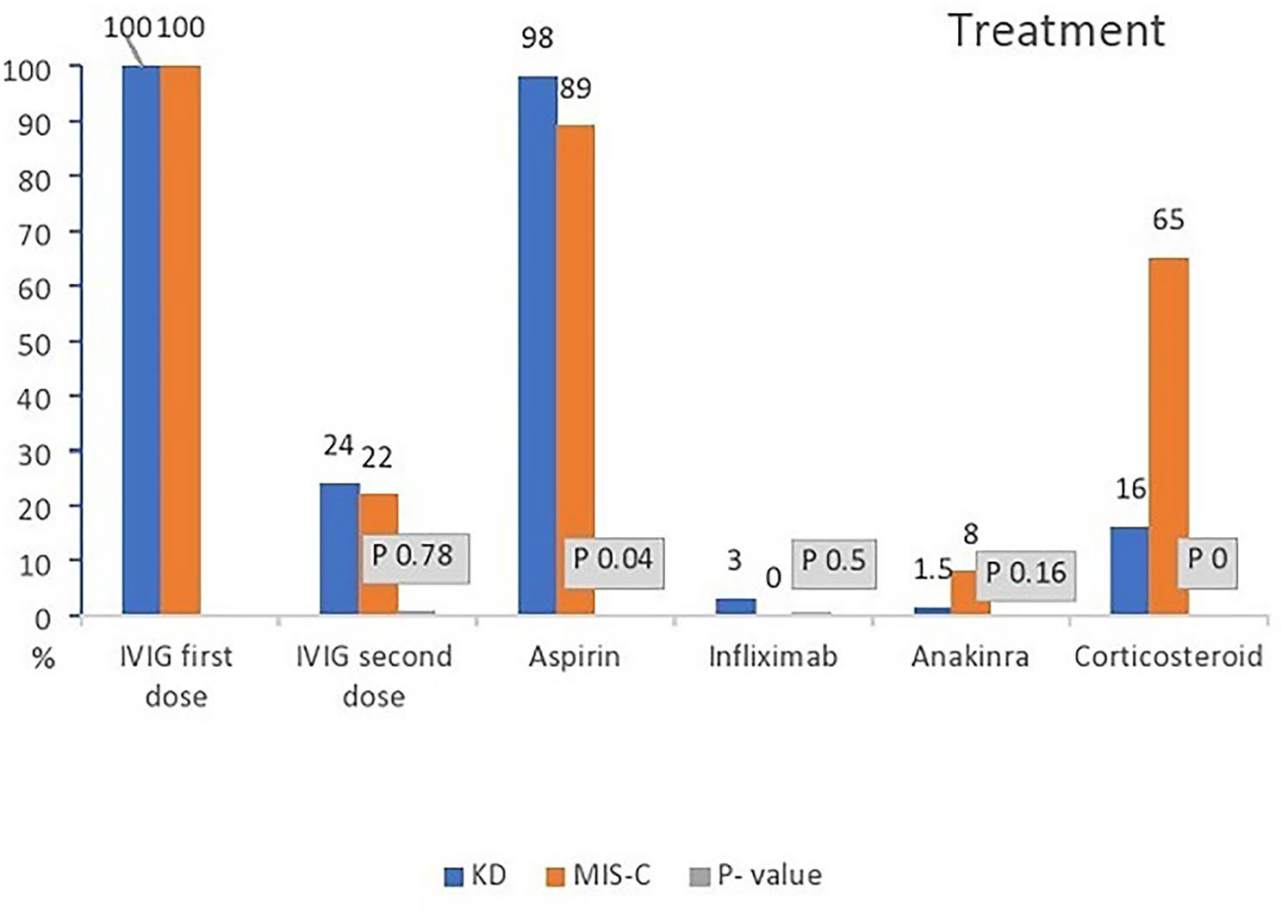
Treatment during admission

A high dose (2 g/kg) of intravenous immunoglobulin (IVIG) was commenced for all patients with KD and MIS-C as the first line of treatment. Figure 2 shows that corticosteroids (methylprednisolone or prednisolone) were given to 65% of patients in the MIS-C group compared with 16% of patients in the KD group (P < 0.001). The use of aspirin was lower in the MIS-C group than in the KD group, 89% vs. 98.5% (P = 0.04). There were no significant differences between the groups in receiving a second dose of IVIG, infliximab, or anakinra. The patients with MIS-C tended to be sicker, and 33% needed admission to the PICU versus 7.5% of KD patients (P < 0.001). In addition, they needed treatment with multiple medications: a second dose of IVIG, anakinra, tocilizumab or corticosteroids (64% in MIS-C, 33% in KD, P < 0.001). However, the duration of stay was similar in both groups, with a median of 5 days. The outcome was good, and all of our patients were discharged (Table 5).

**Table 5:** Outcome

			KD		MIS-C		P-Value
Length of stay, median, IQR, range			5, 2, (2–26)		5, 4, (2–67)		0.52
PICU admission, No (%)			5 (7.5)		18 (33)		**< 0.001**
Multidrug treatment, No (%)			67 (33)		55 (64)		**< 0.001**
Discharge home			67 (100)		56 (100)		

Abbreviation: KD, Kawasaki disease; MIS-C, Multisystem inflammatory syndrome in children; No, number of patients with data; (%), percentage of patients with positive findings; IQR, interquartile range

### A formula differentiating KD from MIS-C

To differentiate between KD and MIS-C, we created a formula using the clinical and laboratory differences between our patients in this study. This formula showed that younger age, along with leukocytosis and thrombocytosis in favor of KD. [age < 7.2 years + WBC > 11.56 (10(3) µcl) + PLT > 236 (10(3) µcl)] = KD; otherwise, patient will be given a MIS-C diagnosis.

To test this formula, we performed a literature review (Supplementary Tables 1, 2) and collected data for patients independently diagnosed with MIS-C (N = 42) or KD (N = 45). Applying our formula above to this control group showed that it was 92% sensitive and 75% specific in detecting MIS-C patients.

## Discussion

Kawasaki disease was first reported by Dr. Tomisku Kawasaki in 1967 [1]. Over the last few decades, KD has been considered one of the leading causes of acquired heart disease in children in the developed world and one of the most challenging diseases in childhood [4]. Extensive research has been done about this illness; however, no definitive causes or diagnostic tests have been identified until now. During this COVID-19 pandemic, there has been an increase in the number of Kawasaki-like cases identified as multisystem inflammatory syndrome in children (MIS-C) [8].

Our descriptive study showed overlap between KD and MIS-C patients in clinical features on admission along with increased inflammatory markers and response to the same treatment. These findings are consistent with a similar hypothesis discussed by Esteve-Sole et al. [8]. Nonetheless, MIS-C has its own unique characteristics, such as affecting older children and presenting with gastrointestinal symptoms. The duration of fever appears to be shorter in MIS-C than in KD [9]. Our hypothesis that this can be explained by the case definition, but it could also be a sign of a more severe illness course for MIS-C that worsens over a short time.

Our study is in line with other published studies. It showed significant lymphopenia and increased procalcitonin and ferritin levels in MIS-C patients compared with KD patients [9]. Additionally, platelets in MIS-C were observed to be low compared with thrombocytosis in KD. Notably, thrombocytopenia has historically been demonstrated to be a sensitive prognostic marker for severe KD [4]. In contrast to the published national data of MIS-C, our study showed no significant difference in CRP and D-dimer between KD and MIS-C [10].

Given that Kawasaki disease has been one of the leading causes of acquired heart disease in children over the last few decades, our study, along with other published studies, refers to MIS-C as the possible new leader [8, 11]. In this study cardiac dysfunction occurred more frequently in MIS-C than in KD and resolved completely after treatment. Interestingly, at the time we reported this study, two of our patients from the MIS-C group with normal echocardiograms on admission showed dilated coronary arteries on follow-up. However, follow-up studies are required to determine the final cardiac consequences of this new disease.

With our findings that MIS-C patients mimic KD with some peculiarities, such as thrombocytopenia and cardiac dysfunction; worsen over a short period; and need intensive care admission, we hypothesize that MIS-C, rather than being its own entity, is a severe manifestation of the KD spectrum. Hence, differentiating between both is vital due to its implications for the management plan.

The formula we created will help. However, we need a larger sample size to test this formula and more variables to increase the sensitivity and specificity. We included only age, WBC, and PLT in this formula, as they were the most available variables in the literature for KD patients.

Timely diagnosis remains a significant challenge for both diseases. The higher incidence of complications within a short period of fever in the MIS-C group makes the diagnosis more challenging, especially when the presentations mimic other diseases. Previous studies showed that starting treatment for KD within the first ten days of fever is vital to decreasing cardiac complications from 25% to 3–5% [12]. For MIS-C, more follow-up studies are needed to evaluate long-term consequences and their relationship with fever duration on presentation and time to intervention. However, treatment should be started as soon as possible in both diseases.

Our study was performed at two pediatric tertiary centers in Dubai, and multiple nationalities were included, but this still does not reflect the diversity of the whole region. Additionally, more studies are needed to follow up with these patients for a more extended period and evaluate similarities and differences in the long-term sequelae of both conditions.

## Conclusion

The COVID-19 pandemic has impacted all aspects of life, especially medicine. As we were trying to understand Kawasaki disease and to identify its causes, a new version has emerged: multisystem inflammatory syndrome in children (MIS-C). MIS-C resembles KD but has new features and a more aggressive clinical course. More studies are needed to further understand both diseases. Long-term follow-up of MIS-C patients is crucial to identifying its effects over time.

## Electronic supplementary material

Below is the link to the electronic supplementary material.

Supplementary Material 1

## Data Availability

All data used in this current study are presented in accompanying tables and figures. Any additional information can be directly obtained from the corresponding author upon reasonable request.

## Abbreviations

MIS-C: Multisystem inflammatory syndrome in children
KD: Kawasaki Disease
No: Number of patients
IQR: Interquartile range
SD: Standard deviation
Y: Years
WBC: White Blood Cells
Hb: Haemoglobin
ANC: Absolute Neutrophils Count
ALC: Absolute lymphocyte Count
PLT: Platelet
CRP: C-Reactive Protein
ESR: Erythrocyte Sedimentation Rate
PCT: Procalcitonin
ALT: Alanine Aminotransferase
AST: Aspartate Aminotransferase
ALB: Albumin
Urine WBC: White blood cell in urine more than 5
Urine RBC: Red blood cell in urine more than 5
Urine protein: Proteinuria
MR: Mitral regurgitation
GI: Gastrointestinal

## References

[1] Gkoutzourelas A, Bogdanos DP, Sakkas LI (2020). Kawasaki Disease and COVID-19. Mediterr J Rheumatol.

[2] Chang LY, Lu CY, Shao PL (2014). Viral infections associated with Kawasaki disease. J Formos Med Assoc.

[3] Rehman S, Majeed T, Ansari MA, Al-Suhaimi EA (2020). Syndrome resembling Kawasaki disease in COVID-19 asymptomatic children. J Infect Public Health.

[4] McCrindle BW, Rowley AH, Newburger JW (2017). Diagnosis, treatment, and long-term management of Kawasaki Disease: A Scientific Statement for Health Professionals from the American Heart Association. Circulation.

[5] Centers for Disease Control and Prevention. Multisyst1. Centers for Disease Control and Prevention. Multisystem Inflammatory Syndrome (MIS). https://www.cdc.gov/mis/index.html. https://www.cdc.gov/mis/index.html. Published 2021. Accessed August 8, 2021.em Inflammatory Syndrome (MIS). https://www.cdc.gov/mis/index.html. https://www.cdc.gov/mis/index.html. Published 2021. Accessed August 8, 2021.

[6] World Health Organization. Multisystem inflammatory syndrome in children and adolescents temporally related to COVID-19. https://www.who.int/news-room/commentaries/detail/multisystem-inflammatory-syndrome-in-children-and-adolescents-with-covid-19. Published 2020. Accessed August 8, 2021.

[7] Maggio MC, Corsello G. Atypical and incomplete Kawasaki disease. 2015. doi:10.1186/1824-7288-41-S2-A45.

[8] Esteve-Sole A, Anton J, Pino-Ramirez RM, et al. Similarities and differences between the immunopathogenesis of COVID-19-related pediatric multisystem inflammatory syndrome and Kawasaki disease. J Clin Invest. 2021;131(6). 10.1172/JCI144554. 10.1172/JCI144554 PMC7954607 33497356

[9] Bar-Meir M, Guri A, Godfrey ME (2021). Characterizing the differences between multisystem inflammatory syndrome in children and Kawasaki disease. Sci Rep.

[10] Bukulmez H. Current understanding of Multisystem Inflammatory Syndrome (MIS-C) following COVID-19 and its distinction from Kawasaki Disease. 1926. doi:10.1007/s11926-021-01028-4/Published. 10.1007/s11926-021-01028-4 PMC8254432 34216296

[11] Pouletty M, Borocco C, Ouldali N (2020). Paediatric multisystem inflammatory syndrome temporally associated with SARS-CoV-2 mimicking Kawasaki disease (Kawa-COVID-19): a multicentre cohort. Ann Rheum Dis.

[12] Tina K. Sosa. Kawasaki Disease: Practice Essentials, Background, Pathophysiology. https://emedicine.medscape.com/article/965367-overview. Published 2018. Accessed August 10, 2021.

