# Supplementary material for: Kawasaki disease (KD) and multisystem inflammatory syndrome in children (MIS-C) in a Middle Eastern patient cohort
Source: Pediatr Rheumatol Online J. 2023 Jun 29;21:64. doi: 10.1186/s12969-023-00834-7 (PMC10308603; doi:10.1186/s12969-023-00834-7)
Supplement: Supplementary file 1 — Supplementary Material 1 [file 12969_2023_834_MOESM1_ESM.docx]

| **Supplementary Table 1. MIS-C associated with COVID-19** | | | |
| --- | --- | --- | --- |
| Organisation or publication | WHO | US Centre for Disease Control and Prevention | Royal College of Paediatrics and Child Health |
| Age group | 0–19 years | <21 years | Child (age not specified) |
| Inflammation | Fever and elevated inflammatory markers for 3 days or more | Fever and elevated inflammatory markers | Fever and elevated inflammatory markers |
| Main features | 1. Rash or bilateral non-purulent conjunctivitis or muco-cutaneous inflammation signs (oral, hands or feet). 2. Hypotension or shock. 3. Features of myocardial dysfunction, pericarditis, valvulitis, or coronary abnormalities (including ECHO findings or elevated Troponin/NT-proBNP), 4. Evidence of coagulopathy (by PT, PTT, elevated d-Dimers). 5. Acute gastrointestinal problems (diarrhoea, vomiting, or abdominal pain). | Clinically severe illness requiring hospitalisation; and multisystem (two or more) organ involvement (cardiac, renal, respiratory, haematological, gastrointestinal, dermatological, or neurological) | Single or multiple organ dysfunction (shock or respiratory, renal, gastrointestinal, or neurological disorder; additional features |
| SARS-CoV-19 | Evidence of COVID-19**(**RT-PCR, antigen test or serology positive), or likely contact with patients with COVID-19. | Other plausible alternative diagnoses Positive RT-PCR, serology, or antigen test; or COVID-19 exposure within the past 4 weeks before symptom onset | RT-PCR positive or negative |
| Exclusion | Other microbial cause of inflammation | Other plausible alternative diagnoses | Any other microbial cause |

| **Supplementary Table 2. All MIS-C cases in this study along with evidence of SARS-CoV-2 infection, fever, and inflammatory markers** | | | | | |
| --- | --- | --- | --- | --- | --- |
| Case ID | Evidence of SARS-CoV-2 | | | **Fever (Duration in days)** | **Raised inflammatory markers (CRP, PCT, ESR)** |
|  | **Exposure** | **Positive antibodies** | **Positive PCR** |  |  |
| MIS-C 1 | Yes | No | No | 5 | yes |
| MIS-C 2 | yes | yes | No | 5 | yes |
| MIS-C 3 | yes | No | No | 6 | yes |
| MIS-C 4 | yes | No | No | 7 | yes |
| MIS-C 5 | yes | No | No | 10 | yes |
| MIS-C 6 | yes | No | No | 4 | yes |
| MIS-C 7 | yes | No | No | 3 | yes |
| MIS-C 8 | yes | No | No | 2 | yes |
| MIS-C 9 | yes | No | No | 4 | yes |
| MIS-C 10 | No | yes | No | 10 | Yes |
| MIS-C 11 | yes | No | No | 6 | yes |
| MIS-C 12 | yes | yes | yes | 5 | yes |
| MIS-C 13 | yes | No | No | 9 | yes |
| MIS-C 14 | yes | No | No | 3 | yes |
| MIS-C 15 | No | yes | yes | 4 | Yes |
| MIS-C 16 | No | yes | No | 10 | Yes |
| MIS-C 17 | yes | No | No | 5 | Yes |
| MIS-C 18 | yes | No | No | 11 | Yes |
| MIS-C 19 | yes | yes | No | 7 | Yes |
| MIS-C 20 | yes | yes | No | 6 | Yes |
| MIS-C 21 | yes | yes | No | 4 | Yes |
| MIS-C 22 | yes | No | No | 12 | Yes |
| MIS-C 23 | yes | yes | No | 6 | Yes |
| MIS-C 24 | yes | yes | No | 3 | Yes |
| MIS-C 25 | No | yes | No | 7 | Yes |
| MIS-C 26 | yes | yes | No | 10 | Yes |
| MIS-C 27 | yes | yes | yes | 5 | Yes |
| MIS-C 28 | yes | yes | No | 7 | Yes |
| MIS-C 29 | yes | yes | No | 4 | Yes |
| MIS-C 30 | yes | yes | No | 6 | Yes |
| MIS-C 31 | yes | yes | No | 5 | Yes |
| MIS-C 32 | No | yes | No | 5 | Yes |
| MIS-C 33 | yes | No | No | 5 | Yes |
| MIS-C 34 | yes | No | yes | 2 | Yes |
| MIS-C 35 | yes | yes | No | 4 | Yes |
| MIS-C 36 | yes | No | yes | 5 | Yes |
| MIS-C 37 | yes | yes | yes | 6 | Yes |
| MIS-C 38 | No | yes | yes | 3 | Yes |
| MIS-C 39 | yes | yes | yes | 4 | Yes |
| MIS-C 40 | yes | yes | yes | 3 | Yes |
| MIS-C 41 | yes | yes | yes | 3 | Yes |
| MIS-C 42 | yes | yes | No | 4 | Yes |
| MIS-C 43 | No | yes | No | 14 | Yes |
| MIS-C 44 | No | yes | No | 9 | Yes |
| MIS-C 45 | yes | No | No | 3 | Yes |
| MIS-C 46 | yes | yes | No | 3 | Yes |
| MIS-C 47 | No | yes | yes | 7 | Yes |
| MIS-C 48 | yes | yes | No | 6 | Yes |
| MIS-C 49 | yes | No | yes | 4 | Yes |
| MIS-C 50 | yes | yes | No | 5 | Yes |
| MIS-C 51 | yes | No | No | 9 | Yes |
| MIS-C 52 | yes | yes | No | 10 | Yes |
| MIS-C 53 | yes | yes | No | 5 | Yes |
| MIS-C 54 | yes | yes | No | 4 | Yes |
| MIS-C 55 | yes | yes | No | 5 | Yes |
| MIS-C 56 | Yes | Yes | Yes | 7 | Yes |

| \| **Supplementary Table 3. All KD cases in this study with symptoms related to KD criteria.** \| \| \| \| \| \| \| \| \| --- \| --- \| --- \| --- \| --- \| --- \| --- \| --- \| \| **Cases with three or less criteria, had abnormal blood tests or echo findings consistent with atypical or incomplete KD.** \| \| \| \| \| \| \| \| \|  \| \| \| **Inflammatory Markers (CRP and/or ESR)**  **High in all cases** \| \| \| \|  \| \| **Case ID** \| **Age**  **(Years)** \| **Fever (Days)** \| **Conjunctivitis** \| **Oropharyngeal Signs** \| **Skin rash** \| **Neck lymphadenitis** \| **Edema of hand and foot** \| \| **KD-1** \| 0.33 \| 15 \| Yes \| Yes \| Yes \| No \| No \| \| **KD-2** \| 0.14 \| 9 \| Yes \| Yes \| Yes \| No \| Yes \| \| **KD-3** \| 1.67 \| 15 \| Yes \| Yes \| Yes \| Yes \| No \| \| **KD-4** \| 2.51 \| 7 \| Yes \| Yes \| Yes \| Yes \| No \| \| **KD-5** \| 7.16 \| 10 \| Yes \| Yes \| Yes \| Yes \| No \| \| **KD-6** \| 1.1 \| 5 \| Yes \| Yes \| Yes \| No \| Yes \| \| **KD-7** \| 0.61 \| 12 \| Yes \| Yes \| Yes \| No \| No \| \| **KD-8** \| 2.3 \| 6 \| Yes \| Yes \| No \| No \| No \| \| **KD-9** \| 3.77 \| 5 \| Yes \| Yes \| No \| Yes \| Yes \| \| **KD-10** \| 2.79 \| 5 \| No \| Yes \| Yes \| No \| No \| \| **KD-11** \| 1.19 \| 7 \| Yes \| Yes \| Yes \| Yes \| No \| \| **KD-12** \| 2.86 \| 10 \| No \| Yes \| Yes \| Yes \| Yes \| \| **KD-13** \| 5.12 \| 12 \| Yes \| Yes \| Yes \| Yes \| Yes \| \| **KD-14** \| 0.52 \| 8 \| Yes \| Yes \| Yes \| No \| Yes \| \| **KD-15** \| 1.7 \| 5 \| Yes \| Yes \| Yes \| No \| Yes \| \| **KD-16** \| 0.74 \| 5 \| Yes \| Yes \| Yes \| No \| Yes \| \| **KD-17** \| 5.54 \| 11 \| No \| Yes \| No \| Yes \| Yes \| \| **KD-18** \| 5.97 \| 5 \| Yes \| Yes \| Yes \| Yes \| Yes \| \| **KD-19** \| 9.06 \| 7 \| Yes \| Yes \| Yes \| Yes \| Yes \| \| **KD-20** \| 2.41 \| 7 \| No \| Yes \| Yes \| No \| No \| \| **KD-21** \| 0.23 \| 5 \| No \| Yes \| Yes \| Yes \| No \| \| **KD-22** \| 0.66 \| 5 \| No \| No \| No \| No \| No \| \| **KD-23** \| 4.01 \| 8 \| Yes \| Yes \| Yes \| Yes \| Yes \| \| **KD-24** \| 0.92 \| 4 \| Yes \| Yes \| Yes \| No \| No \| \| **KD-25** \| 5.02 \| 7 \| Yes \| Yes \| Yes \| Yes \| Yes \| \| **KD-26** \| 5.69 \| 10 \| Yes \| Yes \| No \| Yes \| Yes \| \| **KD-27** \| 0.64 \| 5 \| Yes \| Yes \| Yes \| No \| No \| \| **KD-28** \| 2.89 \| 5 \| Yes \| No \| Yes \| No \| Yes \| \| **KD-29** \| 2.42 \| 4 \| Yes \| Yes \| Yes \| No \| Yes \| \| **KD-30** \| 1.32 \| 8 \| No \| Yes \| No \| No \| No \| \| **KD-31** \| 3.61 \| 5 \| Yes \| Yes \| Yes \| Yes \| Yes \| \| **KD-32** \| 1.1 \| 7 \| No \| No \| No \| No \| No \| \| **KD-33** \| 0.4 \| 14 \| No \| No \| No \| No \| No \| \| **KD-34** \| 10.7 \| 6 \| Yes \| Yes \| Yes \| Yes \| No \| \| **KD-35** \| 0.92 \| 4 \| No \| Yes \| Yes \| No \| Yes \| \| **KD-36** \| 0.92 \| 13 \| No \| No \| No \| No \| No \| \| **KD-37** \| 1.45 \| 5 \| Yes \| Yes \| Yes \| Yes \| Yes \| \| **KD-38** \| 0.31 \| 6 \| No \| No \| No \| No \| No \| \| **KD-39** \| 7 \| 13 \| No \| Yes \| Yes \| Yes \| No \| \| **KD-40** \| 2.75 \| 7 \| Yes \| Yes \| Yes \| No \| Yes \| \| **KD-41** \| 0.7 \| 6 \| No \| Yes \| No \| No \| Yes \| \| **KD-42** \| 0.34 \| 4 \| Yes \| Yes \| Yes \| Yes \| Yes \| \| **KD-43** \| 4.16 \| 5 \| Yes \| Yes \| No \| Yes \| No \| \| **KD-44** \| 2.58 \| 10 \| Yes \| Yes \| Yes \| Yes \| No \| \| **KD-45** \| 1.85 \| 6 \| Yes \| Yes \| Yes \| No \| No \| \| **KD-46** \| 1.17 \| 5 \| No \| Yes \| Yes \| No \| No \| \| **KD-47** \| 2.29 \| 10 \| Yes \| Yes \| Yes \| Yes \| No \| \| **KD-48** \| 9.14 \| 10 \| Yes \| Yes \| Yes \| Yes \| Yes \| \| **KD-49** \| 1.71 \| 5 \| Yes \| No \| No \| Yes \| No \| \| **KD-50** \| 5.87 \| 5 \| No \| Yes \| Yes \| No \| No \| \| **KD-51** \| 6.59 \| 7 \| No \| No \| Yes \| No \| No \| \| **KD-52** \| 3.52 \| 5 \| Yes \| Yes \| Yes \| Yes \| No \| \| **KD-53** \| 1.98 \| 4 \| Yes \| Yes \| Yes \| Yes \| No \| \| **KD-54** \| 0.54 \| 6 \| Yes \| Yes \| Yes \| No \| No \| \| **KD-55** \| 3.86 \| 4 \| Yes \| Yes \| No \| Yes \| No \| \| **KD-56** \| 2.44 \| 7 \| Yes \| Yes \| Yes \| Yes \| Yes \| \| **KD-57** \| 0.44 \| 6 \| Yes \| Yes \| Yes \| No \| Yes \| \| **KD-58** \| 1.76 \| 5 \| Yes \| Yes \| Yes \| Yes \| No \| \| **KD-59** \| 1.59 \| 7 \| No \| Yes \| Yes \| Yes \| Yes \| \| **KD-60** \| 0.52 \| 4 \| Yes \| Yes \| Yes \| No \| No \| \| **KD-61** \| 6 \| 10 \| Yes \| Yes \| No \| No \| No \| \| **KD-62** \| 0.81 \| 4 \| Yes \| Yes \| Yes \| Yes \| Yes \| \| **KD-63** \| 1.08 \| 6 \| No \| No \| Yes \| No \| No \| \| **KD-64** \| 4 \| 7 \| Yes \| Yes \| Yes \| Yes \| Yes \| \| **KD-65** \| 2.73 \| 6 \| No \| Yes \| No \| Yes \| No \| \| **KD-66** \| 4.63 \| 6 \| Yes \| Yes \| Yes \| Yes \| No \| \| **KD-67** \| 6.11 \| 9 \| Yes \| Yes \| Yes \| Yes \| No \| |
| --- | --- | --- | --- | --- | --- | --- | --- | --- | --- | --- | --- | --- | --- | --- | --- | --- | --- | --- | --- | --- | --- | --- | --- | --- | --- | --- | --- | --- | --- | --- | --- | --- | --- | --- | --- | --- | --- | --- | --- | --- | --- | --- | --- | --- | --- | --- | --- | --- | --- | --- | --- | --- | --- | --- | --- | --- | --- | --- | --- | --- | --- | --- | --- | --- | --- | --- | --- | --- | --- | --- | --- | --- | --- | --- | --- | --- | --- | --- | --- | --- | --- | --- | --- | --- | --- | --- | --- | --- | --- | --- | --- | --- | --- | --- | --- | --- | --- | --- | --- | --- | --- | --- | --- | --- | --- | --- | --- | --- | --- | --- | --- | --- | --- | --- | --- | --- | --- | --- | --- | --- | --- | --- | --- | --- | --- | --- | --- | --- | --- | --- | --- | --- | --- | --- | --- | --- | --- | --- | --- | --- | --- | --- | --- | --- | --- | --- | --- | --- | --- | --- | --- | --- | --- | --- | --- | --- | --- | --- | --- | --- | --- | --- | --- | --- | --- | --- | --- | --- | --- | --- | --- | --- | --- | --- | --- | --- | --- | --- | --- | --- | --- | --- | --- | --- | --- | --- | --- | --- | --- | --- | --- | --- | --- | --- | --- | --- | --- | --- | --- | --- | --- | --- | --- | --- | --- | --- | --- | --- | --- | --- | --- | --- | --- | --- | --- | --- | --- | --- | --- | --- | --- | --- | --- | --- | --- | --- | --- | --- | --- | --- | --- | --- | --- | --- | --- | --- | --- | --- | --- | --- | --- | --- | --- | --- | --- | --- | --- | --- | --- | --- | --- | --- | --- | --- | --- | --- | --- | --- | --- | --- | --- | --- | --- | --- | --- | --- | --- | --- | --- | --- | --- | --- | --- | --- | --- | --- | --- | --- | --- | --- | --- | --- | --- | --- | --- | --- | --- | --- | --- | --- | --- | --- | --- | --- | --- | --- | --- | --- | --- | --- | --- | --- | --- | --- | --- | --- | --- | --- | --- | --- | --- | --- | --- | --- | --- | --- | --- | --- | --- | --- | --- | --- | --- | --- | --- | --- | --- | --- | --- | --- | --- | --- | --- | --- | --- | --- | --- | --- | --- | --- | --- | --- | --- | --- | --- | --- | --- | --- | --- | --- | --- | --- | --- | --- | --- | --- | --- | --- | --- | --- | --- | --- | --- | --- | --- | --- | --- | --- | --- | --- | --- | --- | --- | --- | --- | --- | --- | --- | --- | --- | --- | --- | --- | --- | --- | --- | --- | --- | --- | --- | --- | --- | --- | --- | --- | --- | --- | --- | --- | --- | --- | --- | --- | --- | --- | --- | --- | --- | --- | --- | --- | --- | --- | --- | --- | --- | --- | --- | --- | --- | --- | --- | --- | --- | --- | --- | --- | --- | --- | --- | --- | --- | --- | --- | --- | --- | --- | --- | --- | --- | --- | --- | --- | --- | --- | --- | --- | --- | --- | --- | --- | --- | --- | --- | --- | --- | --- | --- | --- | --- | --- | --- | --- | --- | --- | --- | --- | --- | --- | --- | --- | --- | --- | --- | --- | --- | --- | --- | --- | --- | --- | --- | --- | --- | --- | --- | --- | --- | --- | --- | --- | --- | --- | --- | --- | --- | --- | --- | --- | --- | --- | --- | --- | --- | --- | --- | --- | --- | --- | --- | --- | --- | --- | --- | --- | --- | --- | --- | --- | --- | --- | --- | --- | --- | --- | --- | --- | --- | --- | --- | --- | --- | --- | --- | --- | --- | --- | --- | --- | --- | --- | --- | --- | --- | --- | --- | --- | --- | --- | --- | --- | --- | --- | --- | --- | --- | --- | --- | --- | --- | --- | --- | --- | --- | --- | --- | --- | --- |

| **Supplementary Table 4. Control cases with MIS-C** | | | | | |
| --- | --- | --- | --- | --- | --- |
| **ID** | **Age (years)** | **WBC**  **10(3) µcL** | **ALC 10(3) µcL** | **PLT 10(3) µcL** | **References link** |
| MIS-C 1 | 5 | 15.2 | 1.4 | 233 | <https://www.cureus.com/articles/55777-> |
| MIS-C 2 | 15 | 11.9 | 0.4 | 213 | <https://www.cureus.com/articles/55777-> |
| MIS-C 3 | 6 | 5.7 | 1.8 | 268 | <https://www.cureus.com/articles/55777-> |
| MIS-C 4 | 6 | 27.3 | 0.9 | 140 | <https://bmcpediatr.biomedcentral.com/articles/10.1186/s12887-021-02743-8#Tab3> |
| MIS-C 5 | 6 | 19.4 | 0.8 | 80 | <https://bmcpediatr.biomedcentral.com/articles/10.1186/s12887-021-02743-8#Tab3> |
| MIS-C 6 | 3 | 16 | 1.2 | 570 | <https://bmcpediatr.biomedcentral.com/articles/10.1186/s12887-021-02743-8#Tab3> |
| MIS-C 7 | 7 | 9.7 | 0.2 | 105 | <https://bmcpediatr.biomedcentral.com/articles/10.1186/s12887-021-02743-8#Tab3> |
| MIS-C 8 | 7 | 16.9 | 2.1 | 116 | <https://bmcpediatr.biomedcentral.com/articles/10.1186/s12887-021-02743-8#Tab3> |
| MIS-C 9 | 9 | 6.9 | 0.6 | 105 | <https://bmcpediatr.biomedcentral.com/articles/10.1186/s12887-021-02743-8#Tab3> |
| MIS-C 10 | 1 | 24.1 | 6.5 | 900 | <https://bmcpediatr.biomedcentral.com/articles/10.1186/s12887-021-02743-8#Tab3> |
| MIS-C 11 | 10 | 16.1 | 1.45 | 252 | https://www.hindawi.com/journals/cripe/2020/8875987/ |
| MIS-C 12 | 6 | 4.7 | 0.09 | 86 | https://www.hindawi.com/journals/cripe/2020/8875987/ |
| MIS-C 13 | 9 | 20.3 | 2.23 | 243 | https://www.hindawi.com/journals/cripe/2020/8875987/ |
| MIS-C 14 | 9 | 3.7 | 0.57 | 45 | https://www.ncbi.nlm.nih.gov/pmc/articles/PMC8183746/ |
| MIS-C 15 | 12 | 4.46 | 0.98 | 224 | https://www.ncbi.nlm.nih.gov/pmc/articles/PMC8183746/ |
| MIS-C 16 | 2 | 5.6 | 1.7 | 275 | https://www.ncbi.nlm.nih.gov/pmc/articles/PMC8183746/ |
| MIS-C 17 | 10 | 4.17 | 0.73 | 162 | https://www.ncbi.nlm.nih.gov/pmc/articles/PMC8183746/ |
| MIS-C 18 | 11 | 8.4 | 0.45 | 125 | https://www.ncbi.nlm.nih.gov/pmc/articles/PMC8183746/ |
| MIS-C 19 | 15 | 13.8 | 1.2 | 70 | https://www.ncbi.nlm.nih.gov/pmc/articles/PMC8222512/ |
| MIS-C 20 | 2 | 7 | 1.3 | 116 | https://www.ncbi.nlm.nih.gov/pmc/articles/PMC8222512/ |
| MIS-C 21 | 12 | 14.5 | 0.58 | 92 | https://www.ncbi.nlm.nih.gov/pmc/articles/PMC8222512/ |
| MIS-C 22 | 8 | 9.2 | 0.4 | 80 | https://www.ncbi.nlm.nih.gov/pmc/articles/PMC8222512/ |
| MIS-C 23 | 14 | 7.9 | 0.3 | 20 | https://www.ncbi.nlm.nih.gov/pmc/articles/PMC8222512/ |
| MIS-C 24 | 10 | 12.5 | 0.675 | 146 | https://www.ncbi.nlm.nih.gov/pmc/articles/PMC8222512/ |
| MIS-C 25 | 18 | 9.5 | 0.65 | 128 | https://www.ncbi.nlm.nih.gov/pmc/articles/PMC8222512/ |
| MIS-C 26 | 9 | 15.9 | 0.27 | 155 | https://www.ncbi.nlm.nih.gov/pmc/articles/PMC8222512/ |
| MIS-C 27 | 12 | 7.3 | - | 143 | https://www.ncbi.nlm.nih.gov/pmc/articles/PMC8983013/ |
| MIS-C 28 | 13 | 10.9 | - | 80 | https://www.ncbi.nlm.nih.gov/pmc/articles/PMC8983013/ |
| MIS-C 29 | 14 | 10.1 | - | 222 | https://www.ncbi.nlm.nih.gov/pmc/articles/PMC8983013/ |
| MIS-C 30 | 10 | 7.89 | - | 80 | https://www.ncbi.nlm.nih.gov/pmc/articles/PMC8983013/ |
| MIS-C 31 | 6 | 5.35 | - | 229 | https://www.ncbi.nlm.nih.gov/pmc/articles/PMC8983013/ |
| MIS-C 32 | 6 | 4.57 | - | 236 | https://www.ncbi.nlm.nih.gov/pmc/articles/PMC8983013/ |
| MIS-C 33 | 15 | 15.2 | - | 170 | https://pubmed.ncbi.nlm.nih.gov/34087834/ |
| MIS-C 34 | 7 | 3 | - | 70 | https://pubmed.ncbi.nlm.nih.gov/34087834/ |
| MIS-C 35 | 7 | 36.5 | - | 473 | https://pubmed.ncbi.nlm.nih.gov/34087834/ |
| MIS-C 36 | 11 | 10.6 | - | 182 | https://pubmed.ncbi.nlm.nih.gov/34087834/ |
| MIS-C 37 | 3 | 5.56 | - | 152 | https://pubmed.ncbi.nlm.nih.gov/34087834/ |
| MIS-C 38 | 1 | 7.24 | - | 107 | https://pubmed.ncbi.nlm.nih.gov/34087834/ |
| MIS-C 39 | 12 | 4.8 | - | 151 | https://pubmed.ncbi.nlm.nih.gov/34087834/ |
| MIS-C 40 | 6 | 8.66 | - | 42 | https://pubmed.ncbi.nlm.nih.gov/34087834/ |
| MIS-C 41 | 5 | 5.9 | - | 230 | https://pubmed.ncbi.nlm.nih.gov/34087834/ |
| MIS-C 42 | 11 | 3.5 | - | 96 | https://pubmed.ncbi.nlm.nih.gov/34087834/ |

| **Supplementary Table 5. Control cases with KD.** | | | | |
| --- | --- | --- | --- | --- |
| **ID** | **Age**  **(Years)** | **WBC**  **10(3) µcL** | **PLT**  **10(3) µcL** | **References**  **link** |
| KD 1 | 5 | 18.5 | 850 | https://www.ncbi.nlm.nih.gov/pmc/articles/PMC5856969/ |
| KD 2 | 1 | 16.2 | 720 | https://www.ncbi.nlm.nih.gov/pmc/articles/PMC5856969/ |
| KD 3 | 3 | 36 | 380 | https://bmcpediatr.biomedcentral.com/articles/10.1186/s12887-018-1306-5 |
| KD 4 | 4.2 | 32.3 | 467 | http://www.kawasakidisease.kr/?ckattempt=1 |
| KD 5 | 2.5 | 19.4 | 387 | http://www.kawasakidisease.kr/?ckattempt=1 |
| KD 6 | 3.1 | 16.6 | 294 | http://www.kawasakidisease.kr/?ckattempt=1 |
| KD 7 | 5.8 | 36.3 | 333 | http://www.kawasakidisease.kr/?ckattempt=1 |
| KD 8 | 8.8 | 4.8 | 236 | http://www.kawasakidisease.kr/?ckattempt=1 |
| KD 9 | 0.6 | 19.1 | 302 | http://www.kawasakidisease.kr/?ckattempt=1 |
| KD 10 | 6.9 | 9.8 | 220 | http://www.kawasakidisease.kr/?ckattempt=1 |
| KD 11 | 2.8 | 6.6 | 225 | http://www.kawasakidisease.kr/?ckattempt=1 |
| KD 12 | 3.6 | 21.8 | 265 | http://www.kawasakidisease.kr/?ckattempt=1 |
| KD 13 | 4.5 | 12.3 | 260 | http://www.kawasakidisease.kr/?ckattempt=1 |
| KD 14 | 1.9 | 13.3 | 384 | http://www.kawasakidisease.kr/?ckattempt=1 |
| KD 15 | 0.8 | 23.9 | 425 | http://www.kawasakidisease.kr/?ckattempt=1 |
| KD 16 | 2.2 | 14.2 | 329 | http://www.kawasakidisease.kr/?ckattempt=1 |
| KD 17 | 0.7 | 19.2 | 406 | http://www.kawasakidisease.kr/?ckattempt=1 |
| KD 18 | 2.9 | 11.57 | 279 | http://www.kawasakidisease.kr/?ckattempt=1 |
| KD 19 | 10 | 2.52 | 172 | http://www.kawasakidisease.kr/?ckattempt=1 |
| KD 20 | 2.5 | 14 | 304 | http://www.kawasakidisease.kr/?ckattempt=1 |
| KD 21 | 1.2 | 14.42 | 544 | http://www.kawasakidisease.kr/?ckattempt=1 |
| KD 22 | 2.8 | 13.9 | 400 | http://www.kawasakidisease.kr/?ckattempt=1 |
| KD 23 | 0.4 | 19.38 | 487 | http://www.kawasakidisease.kr/?ckattempt=1 |
| KD 24 | 4.8 | 12.4 | 260 | http://www.kawasakidisease.kr/?ckattempt=1 |
| KD 25 | 3.1 | 19.3 | 535 | http://www.kawasakidisease.kr/?ckattempt=1 |
| KD 26 | 5.8 | 11.8 | 443 | http://www.kawasakidisease.kr/?ckattempt=1 |
| KD 27 | 1.1 | 14.5 | 332 | http://www.kawasakidisease.kr/?ckattempt=1 |
| KD 28 | 1.1 | 13.66 | 342 | http://www.kawasakidisease.kr/?ckattempt=1 |
| KD 29 | 1.5 | 10 | 292 | <http://www.kawasakidisease.kr/?ckattempt=1> |
| KD 30 | 1.2 | 14.48 | 257 | <https://www.ncbi.nlm.nih.gov/pmc/articles/PMC8191643/> |
| KD 31 | 0.2 | 17.3 | 368 | [https://www.ncbi.nlm.nih.gov/pmc/articles/PMC8191643/](https://nam12.safelinks.protection.outlook.com/?url=https%3A%2F%2Fwww.ncbi.nlm.nih.gov%2Fpmc%2Farticles%2FPMC8191643%2F&data=05%7C01%7C%7C414c77934a104233642f08da3be37c25%7C84df9e7fe9f640afb435aaaaaaaaaaaa%7C1%7C0%7C637888145496230027%7CUnknown%7CTWFpbGZsb3d8eyJWIjoiMC4wLjAwMDAiLCJQIjoiV2luMzIiLCJBTiI6Ik1haWwiLCJXVCI6Mn0%3D%7C3000%7C%7C%7C&sdata=lpiLf1vaRvN5F4eRb90nwNWlbA99r2CxwmVk4ubAJMA%3D&reserved=0) |
| KD 32 | 0.7 | 11.83 | 304 | [https://www.ncbi.nlm.nih.gov/pmc/articles/PMC8191643/](https://nam12.safelinks.protection.outlook.com/?url=https%3A%2F%2Fwww.ncbi.nlm.nih.gov%2Fpmc%2Farticles%2FPMC8191643%2F&data=05%7C01%7C%7C414c77934a104233642f08da3be37c25%7C84df9e7fe9f640afb435aaaaaaaaaaaa%7C1%7C0%7C637888145496230027%7CUnknown%7CTWFpbGZsb3d8eyJWIjoiMC4wLjAwMDAiLCJQIjoiV2luMzIiLCJBTiI6Ik1haWwiLCJXVCI6Mn0%3D%7C3000%7C%7C%7C&sdata=lpiLf1vaRvN5F4eRb90nwNWlbA99r2CxwmVk4ubAJMA%3D&reserved=0) |
| KD 33 | 1.5 | 11.15 | 195 | [https://pubmed.ncbi.nlm.nih.gov/30985646/ Volume 98 - Issue 15 - p e15009](https://pubmed.ncbi.nlm.nih.gov/30985646/%20Volume%2098%20-%20Issue%2015%20-%20p%20e15009) |
| KD 34 | 10 | 14.88 | 412 | <https://journals.lww.com/md-journal/fulltext/2019/05310/a_case_of_kawasaki_disease_presenting_with.47.aspx> |
| KD 35 | 0.3 | 16.6 | 462 | <https://journals.lww.com/md-journal/fulltext/2019/05310/a_case_of_kawasaki_disease_presenting_with.47.aspx> |
| KD 36 | 0.3 | 28.3 | 980 | <https://pubmed.ncbi.nlm.nih.gov/31145317/> |
| KD 37 | 3 | 39.7 | 395 | <https://journals.lww.com/md-journal/fulltext/2015/05010/kawasaki_disease_mimicking_a_parapharyngeal.21.aspx> |
| KD 38 | 1.6 | 23.12 | 168 | <https://pubmed.ncbi.nlm.nih.gov/29879013/> |
| KD 39 | 0.3 | 23.7 | 450 | <https://pubmed.ncbi.nlm.nih.gov/29848518/> |
| KD 40 | 1 | 8.5 | 240 | <https://bmcinfectdis.biomedcentral.com/articles/10.1186/s12879-021-06101-y> |
| KD 41 | 0.8 | 16.9 | 658 | <https://ped-rheum.biomedcentral.com/articles/10.1186/s12969-021-00643-w> |
| KD 42 | 2 | 11.2 | 577 | <https://jmedicalcasereports.biomedcentral.com/articles/10.1186/s13256-021-03219-0> |
| KD 43 | 0.4 | 23.6 | 588 | <https://www.iomcworld.org/open-access/a-typical-kawasaki-disease-in-an-early-infant-a-diagnostic-challenge-case-report.pdf> |
| KD 44 | 3.5 | 30 | 480 | <https://www.banglajol.info/index.php/AKMMCJ/article/view/24985/16876> |
| KD 45 | 1.2 | 12.8 | 302 | <https://theijcp.org/index.php/ijcp/article/view/306/260> |
